# Epidemics

**Published:** 1856-04

**Authors:** S. H. Dickson

**Affiliations:** Prof. of the Theory and Practice of Medicine in the Medical College of South Carolina


### EPIDEMICS.

BY S. H. DICKSON, M. D.,

Prof. of the Theory and Practice of Medicine in the Medical College of South Carolina.

To discover the laws of propagation and progress in the several forms of disease, would certainly advance the great interests of hygiene. Such knowledge could not fail to suggest relevant efforts at arrest, impediment, and correction or counteraction. It is to be feared, however, that our profession bends too exclusive an attention to the inquiry after causes and modes of action rather than the ascertainment of laws—the true function of scientific investigation, at least in its elementary steps.

Chemistry goes rapidly forward. She concerns herself little with the *why;* she seeks to know the facts, and their uniform occurrence, which denotes the law. Astronomy has reached her present sublime position by the same method. She contents herself with the expression of laws by established formulæ, and reasons, in accordance with them, onward from things known to things unknown.

We speak of diseases familiarly as sporadic and epidemic, and sometimes insist on a distinction which has, so far as I know, never been clearly defined. A board of health announces in a certain city the existence of a given pestilence, and while admitting the unwelcome truth, consoles all those concerned, with the information that there are only a few sporadic cases—it is by no means epidemic. Shortly after another report declares reluctantly that the number of cases has increased and is increasing, and that the disease is now epidemic. Where is the line to be drawn? What proportion of any population may be affected without the change having occurred from sporadic to epidemic?

The word is not used in its etymological meaning on the majority of occasions. It has come to be misemployed among physicians almost universally, to point or refer to some obscure mode or modes of causation. Not content with observing and collating the phenomena, they would indicate, however vaguely, the agency by which these are supposed to be brought about. The superstitious, both ancient and modern, ascribe all to direct interposition of a higher power; the arrows of an incensed Apollo; the wrath of an angry God. Sydenham talks of "telluric emanations;" Prout of a ponderable matter in the atmosphere; Holland hints at animalcular invasion; Mitchell and Cowdell at fungous dispersion. This is all very well; men that think must theorize, and every hypothesis has its uses. Even the wildest conjecture is better than a stagnation that must be infertile. Each of these explanations may apply fairly enough to one or two cases; but it would be easy to show their total insufficiency to account for even a moiety of the observed and recorded facts.

The great evil which needs present correction in our medical reasoning is its premature exclusivism. The truth when arrived at in any supposable case must indeed prove absolutely exclusive, and thrust aside all error; but it is arrogant to assume that we have already attained such absolute truth. In the progress of inquiry we meet with facts that we cannot reconcile. Shall we refuse to recognise any of them for that reason? Surely not. Some diseases known to us only as derivative, and others generally derived and linked together in a regular succession, arise sometimes insulatedly, as if of casual or spontaneous occurrence. If all the apparent facts are real, they will in time be reconciled, and we must not now place in factitious opposition, or deny one because we receive the other. The ordinary course of disease becomes familiar to us. Malarious fevers show themselves in certain well-known localities, and at certain seasons. In dense populations we expect the results of crowd-poisoning. A contagious affection being introduced anywhere will attack those in its neighborhood. The rate of usual progress is also familiar, and observation has enabled us to fix upon an average proportion of attacks and of mortality. But it is well known that there occur remarkable and most impressive exceptions to all these familiar rules, and when any one of our long catalogue of diseases transcends the usual bounds, and assails great numbers or spreads with inordinate rapidity or to an unaccustomed extent, we call it epidemic. But with the use of the term comes almost always the false association. The customarily assigned cause is set aside; an unknown and mysterious agency, telluric, cryptogamic, animalcular, atmospheric, is brought in to account for the novel phenomena. It is this course upon which I am venturing to comment, while I protest against the errors into which it has led us. I am willing to admit the general ignorance of causative agencies in our pathology, if that point be insisted on. What I contend for is, that we should not introduce a new and altogether avoidable source of confusion into inquiries already too difficult and obscure.

To be epidemic implies nothing more than this; that the cause, whatever it may be, of the pestilence, is specially, sometimes almost miraculously, active and efficient. The change is only in degree, not in nature. The sporadic and the epidemic are identical; and not as is often assumed, in contrast and opposition, incompatible. Every disease may become epidemic in the sense of mere multiplication of numbers; not only catarrhal and typhous fevers, dysentery and cholera, but small-pox, and whooping-cough, and jaundice, sun-stroke and furuncle, mesmerism, convulsions and spiritualism; contagious and non-contagious maladies, zymotic and nervous diseases of the fluids and of the solids, morbid alterations of the tissues and morbid activity of the functions. To apply the term epidemic to all these promiscuously, however, as expressive of mysterious causative agency, would be irrational. Each has its own law, which we would do well to ascertain and hold to. A contagious epidemic has spread by contagion; a typhus epidemic by ochlesis and its influences, whatever they may be; a malarious epidemic by miasmatic agency; nervous epidemics by sympathy and imitative and other mental functions morbidly excited. There is no general cause which supersedes the other special causes; a point not seldom taken for granted.

"I do not think it contagious," says a writer on dengue—"I do not think it contagious, *because* its invasion was so sudden and general all over the city, that any attempt to trace it from patient to patient, from house to house, or from quarter to quarter, would utterly fail." In the April number, 1855, of the N. W. Med. and Surg. Journal, there is a report from Prof. N. S. Davis, a most competent observer and able reasoner, upon the recent outbreak of small-pox as epidemic, in Chicago. He said: "The disease had been developed with a rapidity and to an extent not easily explained on the supposition of contagion or simple communicability from one to another. Thus, previous to the 1st January, there had been so few cases in the city that the subject had attracted no attention, only one case having come under the observation of the reporter. Between the 6th and 10th of that month, it had sprung up as if by magic in almost every section of the city, and to some extent among all classes of the people." Further on he said: "Of the twenty cases alluded to as occurring under his own care, only one could be traced to any known exposure, or contact with other cases. They were widely separated from each other, in different sections of the city, and among different classes of people." Prof. Davis then comes to the conclusion, that "it is clearly apparent that the city had been visited by a distinct variolous epidemic influence." Now, it cannot surely be necessary to maintain the contagiousness of small-pox; but if the argument above quoted against the contagiousness of dengue be a sound one, it ought logically to apply here also.

The same argument is pressed in another shape equally illogical. Certain diseases can be spread only by personal communication, contact; whence the word contagion. But it has long since lost this strict etymological meaning, and, like a great mass of other words, is used figuratively. Absolute contact is not required for the communication of many known contagious diseases; small-pox, measles, scarlet fever, whooping-cough, &c., which spread themselves a certain distance around their foci—how far no one has determined. When we commence the inquiry, however, as to the contagiousness of any newly discussed malady, strange to say, the old meaning of the word is brought back upon us, and we are required to prove contact, or personal communication, or at any rate near approach—a thing admitted to be unnecessary—a demand, compliance with which is known to be impossible, with regard to any febrile contagion, Haygarth long since told us impossible as to small-pox—an assertion repeated by Prof. Davis and many others. Even Prof. Hume seems to acknowledge and feel the difficulty, and makes what seems to us a needless effort to bring his cases of yellow fever within a certain number of feet or yards of each other, or of certain common centers.

We literally know nothing of the contingencies which affect or modify the extension of any disease—contagious or not contagious. They vary relatively to the cause itself, whatever that may be; to the surrounding circumstances, of course varying infinitely and to the constitutions of the subjects affected. As to the cause—let us reflect upon the strongly marked difference of energy shown by oxygen in its several allotropic states—passive as in common air, ultra-active in ozone. Telegraphic electricity cannot get over an interval of one-sixteenth of an inch; the electricity of the steam-brush makes an easy leap of sixteen feet, more than three thousand times as far; and we have not yet, I think, measured the reach of the "red artillery of Heaven" —the same identical flash of electricity.

These analogies illustrate our views. It is not pretended that even with their aid we can explain any of the laws we are in search of; at present it suffices to apprehend, acknowledge, and establish them.

There is another point with regard to the character of epidemics which is liable to error and misconception, and therefore deserves our careful study. I allude to their geographical relations. The geographical distribution of animals has received much illustration from the distinguished Prof. Agassiz; the geographical distribution of plants is a familiar topic. I used to enjoy very gratifying opportunities of accompanying on botanical excursions the most amiable and highly gifted among our Carolinian savants. It was very common, upon offering him for inquiry, an unrecognised plant found by the way-side, to hear the remark: "Ah! I see: exotic." Our pathologists never make such an observation. No matter what disease they encounter, cholera, yellow fever, dengue. *Exotic?* No! Indigenous, always *indigenous!*

Logicians require a discrimination between the causa *causans*, the formative or generating cause, and the causa *sine qua non*, the condition without which the first would be inoperative, but which has in itself no productive or generative power. It is the great defect of our professional writings that this latter is so frequently made to play the part of both. Want of drainage and other telluric circumstances observed and imagined; heat, electricity, moisture, and other atmospherical contingencies known and conjectured; these in their varied combinations are alleged to account for everything; to give rise to every species of pestilence. We breed all monsters; we need import none.

In the last London Quarterly Review we have the notice of a work by a professed forester, who in his list of trees native of England includes the sycamore, or great maple. The reviewer sets him right, proving its foreign origin, and fixing, by abundant authority, the time of its introduction into the island. We have among us several trees brought from a distance, which, finding soil and climate congenial, flourish and abound. The "pride of India, melia azedarach;" the paper mulberry; the tallow tree; nay, I know not how many others; the rose, the peach and the nectarine; the crape tree; the camelia; some with a little care, some with none at all, seem fixtures. Our woods are fragrant with the sweet yellow jessamine, the gelseminum sempervirens, which serves my purpose well in illustration of a doubtful example: for Prof. Porcher, in his late address before the Historical Society, gives some very good reasons for supposing this—in contradiction to the present universal belief—to be an exotic. Now, thank heaven, it could not by any possibility be exterminated in its wide extension over our fields and forests. Wheat and the dandelion, the cat and the rat, are they not also imported? Do not weeds and vermin follow us everywhere in our migrations, as well as our cereals, our fruits, and our flowers? Everything, like "the star of empire—westward takes its way." The negro and the yellow fever came both from Africa; to both the climate is congenial; both are likely to remain long here. I wonder whether the time will come when both will be pronounced alike indigenous? History gives the date of their entrance here. The present generation ignores or contradicts history as to the pestilence, and affirms that, as it now grows from our soil, it always did so. As well might the same assertion be made concerning the weeds and vermin which we brought across the sea with us. All the early writers ascribe the yellow fever of this city to importation—infection. This is Lining's view. Old Chalmers, treating of "the diseases of South Carolina," does not include it upon the list. He merely mentions it as resembling what he calls "putrid bilious fever." What an absurdity would be at the present day a treatise on the diseases of Charleston, with the yellow fever omitted! But unfortunately it has become a permanent denizen of the Southern portion of the North American continent. It is fully naturalized; and there are serious reasons for the fear that we may never be able to get rid of it.

The same thing is probably true of dengue or break-bone. Wherever it came from, there are many of us who remember well its first invasion. Yet it also seems naturalized, and has become, like the great maple in England, so familiar that many regard it as "native and to the manor born;" and the direct result of those everlasting and ever present telluric and atmospheric combinations that generate everything in the shape of pestilence.

Cholera is the name of a well-known malady, almost universally admitted to be of Eastern origin. Before 1832, we may affirm it to have been unknown upon this continent. Now-a-days we are liable to it, if, as in Buffalo, we dig ditches along our streets; or if, as in New Orleans, we drink the bad water found in the locality. But why does this digging and drinking give rise now to such effects as did not formerly follow from them? Are soil and water changed? Cholera invaded us, as I

have said, in 1832. It overran the country, and disappeared in 1837. Eleven years after it reappeared, simultaneously, in New Orleans and on Staten Island, a thousand miles apart—coincidently with the arrival of two vessels from Europe, on board of which were subjects ill of it. Since that time it has never left us, and most probably will adhere tenaciously to a soil and climate which it finds but too congenial.

Small-pox springs up among us and spreads unaccountably. So do measles and scarlatina. Perhaps no one will be hardy enough to deny that these are Eastern exotics. Whooping cough and mumps we also suppose to have been imported; but we know not whence, or when, or by whom. It is not too much to say that all these are so fixed upon us, either by an unbroken chain of unavoidable, though invisible, transmission, or by preservation in various fomites, that we cannot hope to throw them off, and may regard ourselves as unhappily independent now of any foreign source of their production.

But let us imagine the fortunate possibility of exterminating any one of them upon this continent. How anxiously would we set about to avoid its resuscitation! If we knew from what quarter of the world it would probably invade us, how assiduously we would guard against its introduction! If we knew the contingencies that might favor its invasion and foster its spread, how earnestly we would set ourselves to correct or counteract them! In some cases we think we know these contingencies, and we establish sanitary regulations accordingly. Boards of health are everywhere appointed; everywhere seek for nuisances, and find them abundantly everywhere. The earth and air of a city must always be polluted; cleanliness of surface and purity of waters where many animals live together are simply impossible—unattainable. More's Utopia will be set up long before the speculation of our hygienists can be realized. There is no town in the world, of five thousand inhabitants, in which there is not filth enough at this moment to account for the presence of yellow fever, or cholera, or any other of the epidemics so unhesitatingly ascribed to neglect or contravention of sanitary considerations. Not to speak of the elemental difficulties, entirely out of our control, and so often pressing upon us and deranging the hygienic internal condition of any locality—such as rains, fogs, heat, calms, storms, and the like—what is to be done with the daily deposit of excrementitious matter polluting the atmosphere, washed down with rains, diffusing itself through the soil, engendering animalculi and fungi, and poisoning the wells? Where is the sewer that keeps itself clean? The City of Philadelphia is proverbially neat—well swept and well watered. But it becomes necessary to prove that she is dirty enough to generate yellow fever for herself, and needs not to be beholden to the Mandarin or any other foreign vessel for it, we have a very nauseous picture offered us of the condition of the drains and docks at that particular part of the Delaware near which this innocent but suspected ship was lying.

Staten Island presents a beautiful aspect to the visitor. On landing near the Quarantine ground he is charmed with the appearance of the villages and surrounding country, from the slopes of whose granite hills all impurities are swiftly carried down into the rapid tides of the Narrows, and find their way quickly into the vast neighboring ocean. Here, too, we are deceived, and this locality, so favorable to the hygienist, is found often to entertain—shall we say *generate!*—yellow fever and cholera. It scarcely adds to our surprise when we remember that the foul and festering metropolis, until this very year regarded as the most filthy on our side of the Atlantic, often escapes these evils, when the neighboring island suffers from their presence.

Savannah, too, built upon a high bluff, with a rapid river below for drainage—and a sandy soil whence all waters may filter away—generated, as we are told, within her very bosom, in 1854, the dreadful unknown cause of yellow fever, which was let loose into the air by excavations. There are few cities, if any, of the same population, to be found in the world, whose surface and soil present so little apparently to be dreaded, as our enterprising, well-governed, and prettily situated sister city of the Southern Empire State.

While, therefore, we pay all practicable attention to hygiene, let us not deceive ourselves into the vain belief of the possible attainment of a degree of purity in any city or large town, that shall be absolutely protective or efficient to repel an epidemic pestilence. Gregory incidentally mentions that "the attendant of the nursery of young pheasants at the Vache in Buckinghamshire informed him that the great secret of securing the health of the young brood is to change their sleeping ground daily. If the locality of the encamping aviary—there were five hundred young birds—was not thus changed, a disease speedily developed itself, which was extensively fatal." Now, let the exclusive hygienist—it is against all exclusivism that I am reasoning—let him reflect upon the impossibility of freshening the air of sleeping rooms night after night in a crowded city; in its streets, lanes, alleys, courts, hovels, closets: let him think of the exhalations (dwelt on by Dr. Wells) adhering to the mattrasses of straw or hair or wool or moss; the feather beds and pillows, the blankets and (even more foul) the cotton "comforts" and other bed clothing, and the woolen and cotton body clothing of the poor, "the great unwashed." Can there be any hope—is there any possibility of purification? "The poor you have always with you;" the unhappy and insalubrious condition of their existence is equally permanent and uniform.

Is it not surprising that pestilence is not equally permanent among them? Are we not driven to infer that here, where the causa *sine qua non* is ever present, their frequent escape from disease is owing to the absence of the causa *causans*, and that our duty is two-fold and not simple? Must we not aim as well to prevent the introduction of the latter, as to diminish the intensity and influence of the former? In the great manufactories of gunpowder, explosions every now and then take place, in spite of the incessant and anxious care to prevent them. How absurd it would be if these failures of precaution should induce a reckless indifference, and if the prohibition of iron-heeled shoes, flint and steel, and of pipes and cigars should be taken off.

Sanitary regulations are universally acceptable; they recommend themselves to every reasonable man, as tending—certainly, though imperfectly—to promote our well-being and comfort. We have become reconciled to their inefficiency, and from habit continue to exercise a blind and consoling faith in them.

But against the second mode of protection—quarantines, namely—there exists a universal and indeed a very plausible prejudice. They also have proved inefficient. They are unfamiliar, and nobody gets used to them. They are exceedingly inconvenient; they are actually oppressive and injurious. Even on the most limited scale, and when their application is indubitably proper and necessary, we submit to them most reluctantly, as an intolerable grievance, as when scarlatina breaks out in a family or boarding school, or small-pox in a town; every imaginable evasion of them is attempted, and every means of concealment practised. Everybody repels and abjures them; they are hated and resisted. Lord Morpeth, Earl of Carlyle, is, in his recent work, loud and frequent in his denunciations of them. But this most philanthropic nobleman, in his own person, conveyed small-pox into Malta. Had he been cruelly kept on board of his vessel at quarantine there for four or five days, as he was very tyrannically elsewhere, he would have escaped the misfortune—a painful one to most men—of having introduced into a healthy community and the house of a friend that most loathsome pestilence.

I am by no means so sanguine as to imagine that the time will ever come when any community shall be subject to its own endemics and local epidemics exclusively. We must take the evil with the good. Iceland may, by her insulation, escape small-pox thirty years, and remain more than sixty without measles.

But a mercantile community must suffer the ills as well as enjoy the benefits of commerce.

The medical journals of the present month, (July,) published in New Orleans, inform us that cholera and yellow fever both exist in that devoted city. The opponents of quarantine find in this sad state of things an argument against its oppressive, and, as they maintain, useless restrictions; and those who believe in the spontaneous origin of both these forms of pestilence are strengthened in their opinions. But surely in a matter of such serious importance we are called upon to institute a deliberate inquiry into the true import of the undeniable facts. Nothing should be taken for granted without due and impartial consideration.

Dr. Fenner ascribes yellow fever to a peculiar intensity of malaria, and affirms that "it never bursts out suddenly" in that city, but that "the endemics fevers, bilious remittent and intermittent, gradually run into the yellow fever type, assuming its features."

In the same number of the same periodical from which I have quoted the above phrases—[*N. O. Med. News & Hosp. Gaz.*]—there is an able paper by Prof. Harrison, commencing with the assertion, unequivocally true as I believe, "that the special cause of yellow fever is utterly unknown;" and proving, beyond all reasonable question, that "it cannot be attributed to marsh malaria." In a pamphlet which I have just received from Dr. McFarlane, this point is further labored, and he seems, indeed, to have made it clear enough, that neither mud, moisture, nor malaria have anything to do with the production of the disease; nay, Dr. McF. claims to have "proved to demonstration that wherever filth, heat, moisture, decomposition, exhalation, and malaria are combined in sufficient concentration to produce disease, there yellow fever cannot exist."

In the same journal, Dr. Barton reiterates his doctrine of the sufficiency of known and obvious contingencies to produce both the forms of pestilence mentioned.

A certain temperature, moisture, and radiation, with disturbance of the original soil, give rise to one; "a great variation of these three elements, but more especially variation of the drying power," generates the other.

These differences do not seem very great, but the results are widely apart. A little nitrogen, carbon, and hydrogen, united in certain proportions, give *hydrophobic*—in certain other proportions, *variolous* poison. Must we take for granted that the diversities of effect are owing merely to diversity of proportion of these known substances—or are we not rather led to the conclusion as inevitable, that there is something new and peculiar superadded in each instance, to which we are to attribute the peculiar qualties?

The contingencies required exist in a hundred places, and have existed hundreds of times in New Orleans, as in the seven years, from 1825 inclusive, without the co-existence of yellow fever. Until 1832, they never produced cholera; they were inefficient to produce it again between 1837 and 1848. We cannot help inferring that there is some causative agent present in New Orleans at this time, specifically capable of bringing on cholera; and as the effect is different, another specific cause competent to produce yellow fever. Whence are these agents derived? Analogy may assist us to answer. Dr. McFarlane informs us of "the appearance of small-pox in the interior town of Thibodeauxville." This disease, as also scarlatina and measles, often arise among us obscurely, or, as it were, spontaneously. Does any one believe them to be indigenous or of native origin? The horse, the rat, several imported grasses and weeds, are met with all around us; we are content to trace them from a foreign ancestry. But we are resolute to claim all diseases met with among us as autochthonous, except the two or three above mentioned. The foreign origin and importation of cholera need not now be discussed. Its material cause is fully naturalized and ready to act all along the valley of the Mississippi. It is doubtful whether it will ever die out from these seats. The contingencies enumerated as causative of yellow fever by Drs. Fenner and Barton, exist annually in a hundred places on the earth's surface where yellow fever is unknown. We may ask them also if yellow fever did not exist during the last two summers in a hundred places, not at all, or not obviously liable to the influence of these contingencies. I myself saw cases of it in localities remarkable for the salubrity of their sparse population; comparatively high and dry, and remote from all explanatory conditions, but the new one of railroad intercourse with an infected city. Was it not reasonable to attribute the new fact of their presence to the new, and only new contingency cognizable? That the cause of yellow fever has been for several years in permanent existence in New Orleans, is perfectly clear. That the supply of it is now adequate to produce the disease, even thus early, is unhappily evident. But this admission does not touch the question of its origin—its foreign derivation. Quarantine cannot prevent in New Orleans the prevalence of the disease, while its domestic supply (preserved from last year, doubtless,) exists. But the yellow fever died out, it seems, in 1824; it may become extinct again; and if it should, then these restrictions may avail. Meanwhile, those other localities which have occasionally suffered, as seems proved, from the importation of a malady which they could not generate, may without censure resort to all means which promise even a temporary or partial immunity from so great an evil.—*Charleston Med. Journal.*

